# Most women living with HIV can deliver vaginally—National data from Finland 1993–2013

**DOI:** 10.1371/journal.pone.0194370

**Published:** 2018-03-22

**Authors:** Inka Aho, Marja Kaijomaa, Pia Kivelä, Heljä-Marja Surcel, Jussi Sutinen, Oskari Heikinheimo, with the FINHIVPREG Study team

**Affiliations:** 1 Department of Infectious diseases, Helsinki University Hospital and University of Helsinki, Helsinki, Finland; 2 Department of Obstetrics and Gynecology, Helsinki University Hospital and University of Helsinki, Helsinki, Finland; 3 National Institute of Health and Welfare, Oulu and Faculty of Medicine, University of Oulu, Oulu, Finland; Massachusetts General Hospital, UNITED STATES

## Abstract

**Introduction:**

Vaginal delivery has been recommended for more than ten years for women living with HIV (WLWH) with good virological control. However, in Europe most WLWH still deliver by cesarean section (CS). Our aim was to assess the rate of vaginal delivery and the indications for CS in WLWH over 20 years in a setting of low overall CS rate.

**Materials and methods:**

This was a retrospective study of all WLWH delivering in Finland 1993–2013. We identified the women by combining national health registers and extracted data from patient files.

**Results:**

The study comprised 212 women with 290 deliveries. Over 35% of the women delivered several children during the study years. During 2000–2013, with consistent viral load monitoring, 80.0% showed HIV viral loads <50 copies/mL in the last measurement preceding the delivery. Altogether 74.5% of all WLWH delivered vaginally and the rate of both elective CS and emergency CS was 12.8% each. For most CSs (63.5%) the indication was obstetrical, for 28.4% it was avoiding HIV transmission and for 0.7% it was mother’s request. In hospitals with less than ten HIV-related deliveries during the study period, the rate of elective CS was higher than in more experienced hospitals (22.7% versus 10.6% [*p* = 0.024]). No perinatal HIV transmissions occurred.

**Conclusions:**

Most WLWH can achieve good virological control and deliver vaginally. This will help them to maintain their future child bearing potential and reduce CS-related morbidity.

## Introduction

Proper management of delivery has played a significant role in the prevention of perinatal HIV infection. In the era preceding the modern combination antiretroviral therapy (cART), elective cesarean section (CS) was proven superior to vaginal delivery in preventing perinatal HIV infection [1–3]. Thus, CS became the dominant mode of delivery among HIV-infected parturients in several Western countries [4].

Once the HIV viral load measurement became available, the maternal viral load prior to delivery proved to be the most significant risk factor of perinatal HIV infection. At the same time, implementation of cART as the standard of care during pregnancy lowered the perinatal HIV infection rates, to less than two percent [5]. With such a low transmission rate, the risk reducing effect of CS was no longer evident. This led to revision of several European guidelines to promote vaginal delivery among women with well controlled HIV infection [6].

At present, the threshold to recommend vaginal delivery varies from the 50 copies/mL in the British HIV association (BHIVA) [7] and the European AIDS Clinical Society (EACS) [8] guidelines to that of 400 copies/mL in France [9] and 1000 copies/mL in the Department of Health and Human Services (DHHS) guideline in the US [10]. In Finland, the clinical practice from the early 2000s has been to recommend vaginal delivery in HIV-infected mothers if the viral load is <1000 copies/mL. In 2010, the threshold was lowered to 200 copies/mL [11].

The transition from CS to vaginal delivery in clinical practice has been slow; 40–66% of women living with HIV (WLWH) in Europe, Canada and the US still deliver by CS and 22–51% by elective CS despite excellent virological treatment results [4, 12–17].

As in all Nordic countries, vaginal delivery has been strongly encouraged for the Finnish general population. The overall rate of cesarean delivery has remained in the range of 16–17% during the 2010s [18]. The rate has been similar in Sweden and Norway, but slightly higher in Denmark (22%) [19]. Conversely, regarding the low rate of CS in the general population, a recent study from Denmark concerning WLWH 2002–2014 reported a high rate (67%) of CS [20].

The aim of this study was to determine the national rate of vaginal delivery and the indications for CS in HIV-related pregnancies in a setting of free HIV care and low CS rate in the general population, and to assess whether the recommendations on the mode of delivery were followed.

## Materials and methods

We included all women with at least one delivery in Finland after being diagnosed with HIV infection during 1993–2013.

In Finland, each individual receives a unique, 10-digit personal identification number at birth or immigration by the Civil Registration system. With this number, a person can be identified in different registers and hospital files throughout the country.

Laboratories report each individual’s first positive HIV antibody test to the National Infectious Diseases Register held by the National Institute of Health and Welfare [21]. Physicians report detailed information on mode of transmission of HIV, nationality, country of transmission, and stage of the disease to the registry.

Hospitals report all children born in Finland to the Medical Birth Register regardless of mother’s nationality [18]. This register contains information on the mother, the delivery, and the newborn.

The Finnish Maternity Cohort Register contains information on antenatal infection screening results [22]. The nationwide opt-out screening of HIV was implemented in 1998.

We combined the National Infectious Diseases Register with Medical Birth Register and Finnish Maternity Cohort Register to identify women delivering in Finland after having received HIV-diagnosis. Data on deliveries were collected from the registers as well as from the hospitals’ patient files. The study subjects were not contacted.

We extracted the following data concerning each delivery: ART status, CD4 lymphocyte count at the beginning of the pregnancy, HIV viral load closest preceding the delivery (usually at gestational week 36), mode of delivery, indication for CS, and time from rupture of the membranes to the delivery (ROM).

Mode of delivery was classified as vaginal, elective CS and emergency CS. CSs were classified as elective when taking place before the onset of contractions and before ROM. All other CSs were classified as emergency procedures regardless of the indication.

HIV-viral load monitoring was implemented in 1996, but the data on viral load were inconsistent during 1996–1999. Analyses on viral loads and their relatedness to the mode of delivery were restricted to the years from 2000 onwards when consistent viral load data were available. During the study years, the limit of detection varied from 1000 copies/mL to 20 copies/mL. Since 2001 the limit of detection has been 50 copies/mL or less.

### Ethical approval

The ethics committee of Helsinki University Hospital approved the study (February 10, 2015 344/13/03/00/2014). The National Institute of Health and Welfare granted permission to combine the registers and to perform the nationwide study (December 16, 2015 THL/1535/6.02.00/2015). All participating 18 hospitals provided local permissions to carry out the study and to use their files. According to the Finnish legislation informed consent is not required for this type of retrospective study.

### Statistical analysis

We grouped the viral loads preceding delivery into 4 categories: 1) Viral load undetectable with a threshold used at the time of the measurement; 2) detectable at 50–399 copies/mL; 3) detectable at 400–999 copies/mL; and 4) detectable at ≥1000 copies/mL.

For comparisons between groups, Chi square and Fisher’s exact test were used for categorical variables and the nonparametric Mann–Whitney U-test for continuous variables.

In all statistical analyses IBM SPSS version 21.0 (Chicago, IL, USA) was used.

## Results

The study comprised 212 women with altogether 290 children including four pairs of twins. Of these 212 women, 138 (65.1%) were immigrants, 73 (34.3%) were born in Sub-Saharan Africa. At the time of conception the median age of the women was 30 (interquartile range [IQR]) 26–34), 65.9% were aware of their HIV-infection, 35.9% were on ART, and median CD4 count during the pregnancy was 478 (IQR 339–665) cells/μL. Of these 212 women, 38.2% had delivered before the HIV diagnosis. After the HIV diagnosis 63.2% delivered one child, 31.6% delivered two, and 5.1% delivered three or more children. No perinatal HIV transmissions occurred.

**Fig 1:**
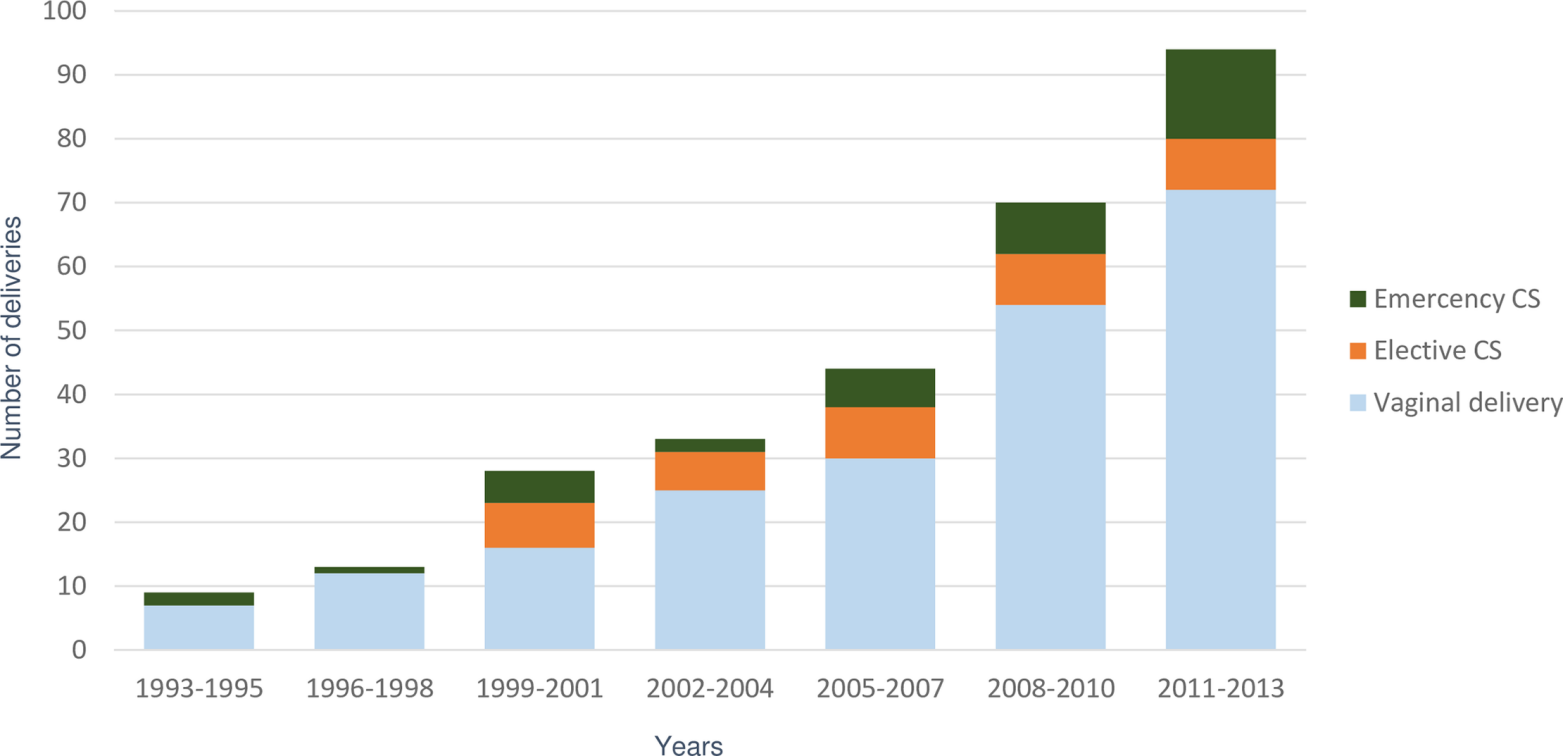
The number and mode of the deliveries among HIV-infected women in Finland 1993–2013.

The annual number of deliveries among HIV-infected women was very small during the early years of the epidemic (2 in 1993) but increased to 38 in 2013. More than half of all the deliveries occurred after 2007 (Fig 1). Helsinki University Hospital accounted for 65% of all the deliveries and three subsequent hospitals five percent each. Five or less HIV-infected women delivered in ten hospitals during the 20 year period.

The overall rate of vaginal delivery was 74.5% and that of both elective and emergency CS 12.8%. Of all vaginal deliveries, 2.8% were assisted vaginal deliveries. Of all deliveries, 83.8% were planned to be vaginal.

The rate of vaginal delivery was 72.7% in the first HIV-associated delivery and 82.2% in subsequent deliveries (p = 0.071). For this same comparison, the rate of emergency CS declined from 14.8% to 4.1% (*p* = 0.013), but the rate of elective CS did not differ, 12.4% versus 13.7%.

In a total of 22 deliveries during 1993–1998, when the mode of delivery was chosen on obstetrical indications only, the rate of vaginal delivery was 86.4%. The remainder were all emergency CSs. During 1999–2013, the annual rate of vaginal delivery varied from 45.5% in 2000 to 95.8% in 2012 (Fig 1).

During 1993–1998, all 22 women received mono- or dual-ART during the last 4–12 weeks of pregnancy. Of them, seven started treatment in the second trimester and one was on medication throughout the pregnancy. Nobody refused ART. Since 1999, in all 265 pregnancies, all women have been offered cART; six women refused ART altogether and two accepted only zidovudine monotherapy. The reasons for declining one or more antiretroviral agents included fear of detrimental effects of ART to the fetus and not believing in modern medication.

Viral load monitoring at gestational week 34–38 was consistent throughout the country during 2000–2013. During this time period, 80.0% of the women achieved undetectable viral load before the delivery and 78.8% of them delivered vaginally (Table 1).

**Table 1:** Mode of the delivery according to HIV viral load 2000–2013.

Mode of delivery	VL BLD	VL 50–399	VL 400–999	VL ≥1000	TOTAL
	*n* = 208	*n* = 30	*n* = 7	*n* = 15	N = 260
**Vaginal**	164 (78.8)	23 (76.7)	2 (28.6)	2 (13.3)	191 (73.5)
**Elective CS**	18 (8.7)	5 (16.7)	2 (28.6)	8 (53.3)	33 (12.7)
**Emergency CS**	26 (12.5)	2 (6.7)	3 (42.9)	5 (33.3)	36 (13.8)

CS, cesarean section; VL, viral load; BLD, viral load below the limit of detection at the time of the measurement. Data are reported as n (%). VLs are reported as copies/mL.

Altogether 37 women delivered with elective CS during 1993–2013 (Table 2). HIV was the most common indication (40.5%) followed by breech presentation (13.5%) and previous CS (8.1%). In 0.7% of all deliveries, the indication for elective CS was mother’s request without virological or obstetrical indication.

**Table 2:** Indications for elective and emergency cesarean sections in all deliveries 1993–2013.

**Indication for elective CS**	**N = 37**	**% of elective CS**	**% of all deliveries**
HIV	15	40.5	5.2
Breech presentation	5	13.5	1.7
Previous CS	3	8.1	1.0
Hepatitis C^a^	2	5.4	0.7
Suspected fetal asphyxia	3	8.1	1.0
Mother’s request	2	5.4	0.7
Acute bleeding	2	5.4	0.7
Other	5	13.5	1.7
**Indications for emergency CS**	**N = 37**	**% of emergency CS**	**% of all deliveries**
Suspected fetal asphyxia	11	29.7	3.8
Failure to progress	11	29.7	3.8
HIV	6	16.2	2.1
Breech presentation	5	13.5	1.7
Previous CS	3	8.1	1.0
Other	1	2.7	0.3

CS, cesarean section.

^a^ At the time of these deliveries (2006–2009) HIV/HCV–coinfection was an indication for elective CS in some hospitals regardless of the HIV-viral load.

Of the 37 emergency CSs, the most common indications were suspected fetal asphyxia and failure to progress, 29.7% each (Table 2). In nine cases the women went into labour before scheduled CS, all turned into emergency CS.

During 1999–2013, when poorly controlled HIV itself was an indication for CS, the proportion of HIV-related CS of all 264 deliveries was (21) 7.8%. This proportion decreased from 11.4% in 1999–2007 to 5.5% in 2008–2013 (*p* = 0.065). Of these 21 HIV-infections, 14 (66.7%) were diagnosed either before the pregnancy or before gestational week 20. Of these 14 women, eight refused cART and six were poorly adherent to ART and failed to suppress viremia. The median (IQR) viral load preceding HIV induced CS was 2530 copies/ml (399–13724).

The volume of deliveries among WLWH impacts on rate of vaginal deliveries. In hospitals having delivered ≥10 HIV-infected parturients, the rate of vaginal delivery varied from 70.0% to 76.5%. Moreover, the rate of elective CS in these hospitals was significantly lower than in the hospitals having delivered <10 HIV-infected parturients (10.6% versus 22.7% [*p* = 0.024]).

The time from rupture of the membranes (ROM) was available for 253 out of 268 of vaginal deliveries during 1999–2013. Median (IQR) time from ROM was 1 (0–7) hour. Time from ROM was more than 24 hours in 2.8% and more than 48 hours in 0.8%.

## Discussion

Of all the deliveries of WLWH in Finland during 1993–2013, 74.5% were vaginal. To our knowledge, this is the highest rate of vaginal delivery of WLWH on a national level of an industrialized country published so far. However, as 83–84% of all deliveries are vaginal in Finland [18], a difference still remains between WLWH and their HIV negative counterparts regarding the mode of delivery.

Our results on the mode of delivery in WLWH differ from those previously published from several well-resourced countries. So far the highest annual rates of vaginal delivery in WLWH have been reported from Canada (65%) and France (53%) [12, 13]. Denmark resembles Finland both in HIV treatment being easily accessible and free of charge, and in low prevalence of CS in the general population. Despite these similarities, the rate of vaginal delivery among HIV-infected women in Finland is more than two-fold higher compared to that seen in Denmark (75% versus 33%) [20].

In the UK, the rate of vaginal delivery among HIV-infected women increased from 23% during 2000–2006 to 36% during 2007–2011 [15]. Even higher increase from 17% to 52% was observed when assessing the mode of delivery before and after revisions of the European national guidelines in a pooled analysis of Swiss Mother & Child HIV Cohort Study and the European Collaborative Study [16]. Converse to these European figures, Kourtis et al. [23] reported that the rate of vaginal delivery among WLWH in the US continued to decline from 50% in 2005 to 42% in 2010 with over 90% of all CSs being elective.

This difference in the rate of vaginal delivery cannot be explained by the HIV treatment results. The proportion of women fulfilling the virological criteria for vaginal delivery in Finland is comparable to that reported from France, Denmark and the US [13, 17, 20]. However, whilst over 75% of women fulfilling these criteria in Finland deliver vaginally, only half of them in France or the US do so [13, 17]. This rate was not reported in the Danish study [20].

In small hospitals with less than ten HIV-related deliveries during the study years, the rate of elective CS was higher than in more experienced hospitals (23% versus 11%). This might imply that centralizing HIV-related deliveries would further decrease the CS rate. However, because of long geographical distances, this is not always feasible. On the other hand, when comparing these rates to the rates of elective CS reported from elsewhere, they are still low [13, 17, 20].

In our national cohort most CSs were performed on obstetrical indications. There was a strong aim towards vaginal delivery already during the early years of the epidemic in Finland with very few CSs in 1993–2000. This might explain, in part, the low rate of CS performed because of a previous CS (11% of all CSs) compared to Denmark (33%), France (32%), and the US (33%) [13, 17, 20]. Moreover, several women delivered vaginally after a previous CS. Thus, vaginal birth after previous CS can be encouraged also among HIV-infected women.

High HIV-viral load was the main indication for CS in only 7% of all deliveries and 28% of all CSs. The main reason for failure to suppress the viremia was not a late diagnosis, since 67% of them were diagnosed either before the pregnancy or before gestational week 20, but poor adherence to HIV-management or complete refusal of cART. In addition to this complete refusal, fear of ART might have accounted for poor adherence, since several women were adherent to appointments but not to ART. These reasons should be evaluated using a multidisciplinary approach, for example mother’s potential concerns of fetal safety of cART should be addressed appropriately. Since today all HIV-infected people are recommended to start cART regardless of their CD4 count, it is important to discuss the choice and safety of medication during pregnancy already before the conception. The best way to prevent HIV-diagnoses during late pregnancy is to continuously offer universal HIV-screening to all women during early pregnancy.

Maternal HIV-infection may have also contributed to the higher rate of emergency CS for fetal asphyxia. The obstetrician may be more likely to proceed to emergency CS in the context of a non-reassuring fetal heart rate tracing, since monitoring of the fetus is difficult when mother-fetal blood contact and fetal blood samples are to be avoided. The same reasoning may account for the high rate of “failure to progress” as an indication for emergency CS. During the study years, a prolonged time after the ROM was considered to increase the perinatal transmission risk and emergency CS was recommended with prolonged time from ROM. A similar phenomenon was reported from Denmark [20]. Since three studies [24–26] have reported that duration of ROM does not affect perinatal transmission risk when maternal viral load is undetectable, the rate of emergency CS will most certainly decrease.

Maternal request without any obstetrical or virological indication for CS was an indication only in 1% of all deliveries. On the contrary, four women refused CS despite viral load >400 copies/mL. These findings are in contrast to Denmark where 40% of women living with HIV still requested elective CS [20].

CS is associated with an increased risk of both maternal and infant morbidity, and these risks are magnified in repeated CSs. Most common maternal short-term complications arise from infections and thromboembolism [27]. Moreover, HIV seems to increase the rates of these short-term complications such as infection, surgical trauma, hospital deaths, and prolonged hospitalization, even in the era of cART [23].

In subsequent pregnancies increased risk of abnormal placentation due to a previous CS, resulting in haemorrhage, blood transfusion and hysterectomy, is responsible for most long-term morbidity. In addition, the risk of prematurity and small-for-gestational-age infant are increased in subsequent pregnancies [27]. These long-term complications have not been studied in HIV-related pregnancies. However, an increasing proportion of women living with HIV desire to have several children [28, 29]. This was also true in our study, where 37% of women had more than one child after being diagnosed with HIV. Thus, CS may lead to increased morbidity in subsequent pregnancies and even limit the possibility of multiple pregnancies.

The major limitation of our study is the small number of HIV-related pregnancies in Finland and the retrospective nature of the study. We were, however, able to use nationwide data and include all WLWH delivering in Finland from the beginning of the epidemic. In addition to registry data, we also had access to actual patient files allowing for such clinically meaningful data as indication for CS.

In conclusion, our study strengthens the evidence that most pregnant women living with HIV in a well-resourced country can achieve low level viremia and deliver vaginally. This is likely to reduce CS-related morbidity and protect future child bearing possibilities.
